# Sleep quality among ICU workers

**DOI:** 10.1186/cc12445

**Published:** 2013-03-19

**Authors:** G Burghi, I Serkochian, B Frache, P Alzugaray, K Goinheix, M Rodriguez Verde, H Bagnulo, E Azoulay

**Affiliations:** 1 Hospital Maciel, Montevideo, Uruguay; 2 Sanatorio Americano, Montevideo, Uruguay; 3 Hospital de Paysandú, Uruguay; 4 Hôpital Saint Louis, Paris, France

## Introduction

Prolonged shifts, workload, stress, and different conflicts are associated with burnout, loss of psychological wellbeing, and probably with an inadequate sleep quality (ISQ). This relevant disturbance leads to deterioration of the work performance, may impair quality of care provided to patients and increases the incidence of serious adverse events. The objective was to determine the prevalence of ISQ and sleepiness among Uruguayan ICU workers, and to evaluate risk factors associated with ISQ.

## Methods

A survey was conducted in six Uruguayan ICUs. The sleep quality was evaluated on the basis of the Pittsburgh score (PS), and the sleepiness was identified by the Epworth scale. ISQ was defined as PS greater than 5 points and sleepiness by an Epworth scale higher than 6 points. ICU's, patient's, and clinician's characteristics were assessed for their association with the prevalence of ISQ. All variables with *P *0.2 in univariate analysis were included in a model of ordinal regression. *P *0.05 was considered statistically significant.

## Results

The survey was completed by 129 ICU workers. The global prevalence of ISQ in ICU was 67.4%. ISQ was observed in 45% of physicians and 82% of nurses and nurses assistant (*P *0.001). Sleep medication was used by 13.3% of the ICU team. Univariate analysis showed that ISQ was significantly associated with sex (73% vs. 43%, *P *= 0.03 in women and men, respectively), marital status (84% vs. 61%, *P *= 0.01 in single and couple workers, respectively), more than 60 hours working in the last week (76% vs. 61%, *P *= 0.07) and less than 6 sleeping hours (95% vs. 54%, *P *0.0001). Multivariable analysis demonstrated that a sleep duration less than 6 hours was independently associated with ISQ (OR = 24.5; 95% CI = 5.2 to 115.8; *P *0.0001). Furthermore, pathologic sleepiness was present in 59.3% of ICU workers. Sleepiness was independently associated with use of sleep medication (OR = 5.9; 95% CI = 1.2 to 28.5; *P *= 0.025).

## Conclusion

The prevalence of ISQ and sleepiness is very high among ICU workers. Those disturbances are independently associated with a sleep duration less than 6 hours, and sleep medication use, respectively. These results highlights that strategies to decrease ISQ and sleepiness in ICU clinicians are urgently needed to improve work performance, improve quality of care provided and prevent adverse events.

